# Effect of Substrate Stiffness on Early Mouse Embryo Development

**DOI:** 10.1371/journal.pone.0041717

**Published:** 2012-07-31

**Authors:** Kevin S. Kolahi, Annemarie Donjacour, Xiaowei Liu, Wingka Lin, Rhodel K. Simbulan, Enrrico Bloise, Emin Maltepe, Paolo Rinaudo

**Affiliations:** 1 Department of Obstetrics Gynecology and Reproductive Sciences, University of California San Francisco, San Francisco, California, United States of America; 2 Department of Pediatrics, University of California San Francisco, San Francisco, California, United States of America; Konkuk University, Republic of Korea

## Abstract

It is becoming increasingly clear that cells are remarkably sensitive to the biophysical cues of their microenvironment and that these cues play a significant role in influencing their behaviors. In this study, we investigated whether the early pre-implantation embryo is sensitive to mechanical cues, i.e. the elasticity of the culture environment. To test this, we have developed a new embryo culture system where the mechanical properties of the embryonic environment can be precisely defined. The contemporary standard environment for embryo culture is the polystyrene petri dish (PD), which has a stiffness (1 GPa) that is six orders of magnitude greater than the uterine epithelium (1 kPa). To approximate more closely the mechanical aspects of the *in vivo* uterine environment we used polydimethyl-siloxane (PDMS) or fabricated 3D type I collagen gels (1 kPa stiffness, Col-1k group). Mouse embryo development on alternate substrates was compared to that seen on the petri dish; percent development, hatching frequency, and cell number were observed. Our results indicated that embryos are sensitive to the mechanical environment on which they are cultured. Embryos cultured on Col-1k showed a significantly greater frequency of development to 2-cell (68±15% vs. 59±18%), blastocyst (64±9.1% vs. 50±18%) and hatching blastocyst stages (54±25% vs. 21±16%) and an increase in the number of trophectodermal cell (TE,65±13 vs. 49±12 cells) compared to control embryos cultured in PD (mean±S.D.; p<.01). Embryos cultured on Col-1k and PD were transferred to recipient females and observed on embryonic day 12.5. Both groups had the same number of fetuses, however the placentas of the Col-1k fetuses were larger than controls, suggesting a continued effect of the preimplantation environment. In summary, characteristics of the preimplantation microenvironment affect pre- and post-implantation growth.

## Introduction

Virtually every living structure, from cells to tissues and up to the level of the entire organism has the capability to sense and respond to physical forces; this property is defined as mechanotransduction. For example, stem cells sense the stiffness of the environment and initiate alternate differentiation patterns [Bibr pone.0041717-Titushkin1] [Bibr pone.0041717-DuFort1] [1–3].

While multiple groups are studying the molecular mechanisms mediating the mechanotransduction effects, relatively few studies have evaluated the role of mechanotransduction during early embryo development [4]. This is important, as events occurring during this sensitive period of development can have long-lasting health effects, as proposed by the Developmental Origins of Health and Disease (DOHaD) Hypothesis [Bibr pone.0041717-Salonen1] [5,6]. For example, preimplantation embryos cultured *in vitro* manifest alterations in gene expression and show abnormal placentation and postnatal growth patterns [Bibr pone.0041717-Rinaudo1] [Bibr pone.0041717-Giritharan1] [Bibr pone.0041717-DellePiane1] [7–10].

**Figure 1:**
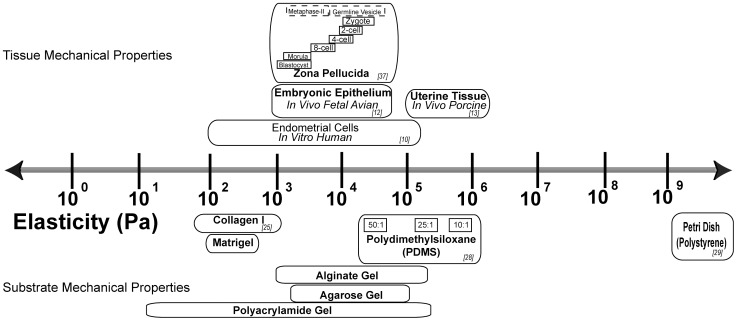
The stiffness or elasticity of various epithelial tissues is compared with the elasticity of materials typically employed in tissue culture.

Particularly striking is the fact that a petri dish, routinely used to grow preimplantation embryos is, at 1 GPa, roughly six orders of magnitude stiffer than the uterine epithelium [Bibr pone.0041717-Thie1] [11,12]. A number of works have demonstrated that embryonic blastomeres, fallopian tube and uterine epithelium are all soft, on the order of 100–1000 Pa [Bibr pone.0041717-Thie1] [Bibr pone.0041717-Filas1] [11,13,14] (**Figure 1**). Since increasing evidence underscores the importance of the mechanical environment in determining cell fate and development, our primary aim was to determine the significance of the mechanical environment in the context of embryo development. We therefore performed experiments with the intent to recapitulate as closely as possible the *in vivo* mechanical conditions of the fallopian tube and uterus. In particular, we aimed to investigate how the mechanical properties (i.e. stiffness of the dish) of the preimplantation embryo culture environment affected blastocyst development and early intrauterine growth.

Investigators have studied whether changes in environment may have a positive effect on embryo development [Bibr pone.0041717-Eaton1] [15,16]. Mechanical stress appears to be necessary, as zero gravity and micro-gravity have proven to be lethal or detrimental to preimplantation embryos [Bibr pone.0041717-CrawfordYoung1] [17,18]. Interestingly, continuous tilting of embryos during culture results in a uniform shear stress and improves development of mouse and human pre-implantation embryos [19]. Evidence from co-culture experiments, in which embryos are grown over a layer of supporting cells (therefore a “softer” environment), is inconclusive [20]. Some authors have attributed the positive effect of coculture to the direct contact between the embryo and the underlying cells [21], in support for culturing embryos on a softer surface. Other authors found that embryo encapsulation with alginate or agarose increase blastocyst development [22], while others have found no effects on blastocyst development or quality [Bibr pone.0041717-Krentz1] [23,24]. A number of studies have shown that the presence of the zona-pellucida (ZP) improves embryo survival in the face of mechanical stress [25].

Materials with different stiffnesses were initially tested for their amenability to embryo culture; polyacrylamide gels, agarose gels, alginate gels, PDMS and collagen gels. Here we report results following preimplantation embryo culture following PDMS or collagen I. We chose to investigate the collagen I gels, because collagen is a component of the uterine extracellular matrix and its elasticity accurately recapitulates the physiological range in uterine environment elasticity [Bibr pone.0041717-Thie1] [11,14]. Furthermore, collagen allowed fabrication of extremely soft gels in the range of ∼1 kPa [26].

We found that culture on softer surface resulted in an increase in 2-cell, blastocyst, and hatching frequency and TE cell number. In addition, fetuses derived from embryos that had been cultured on collagen *in vitro* had a greater placental weight at E12.5.

## Results

### The Stiffness of the PDMS Substrate Affected Hatching Frequency and Total Blastocyst Cell Number

**Figure 2:**
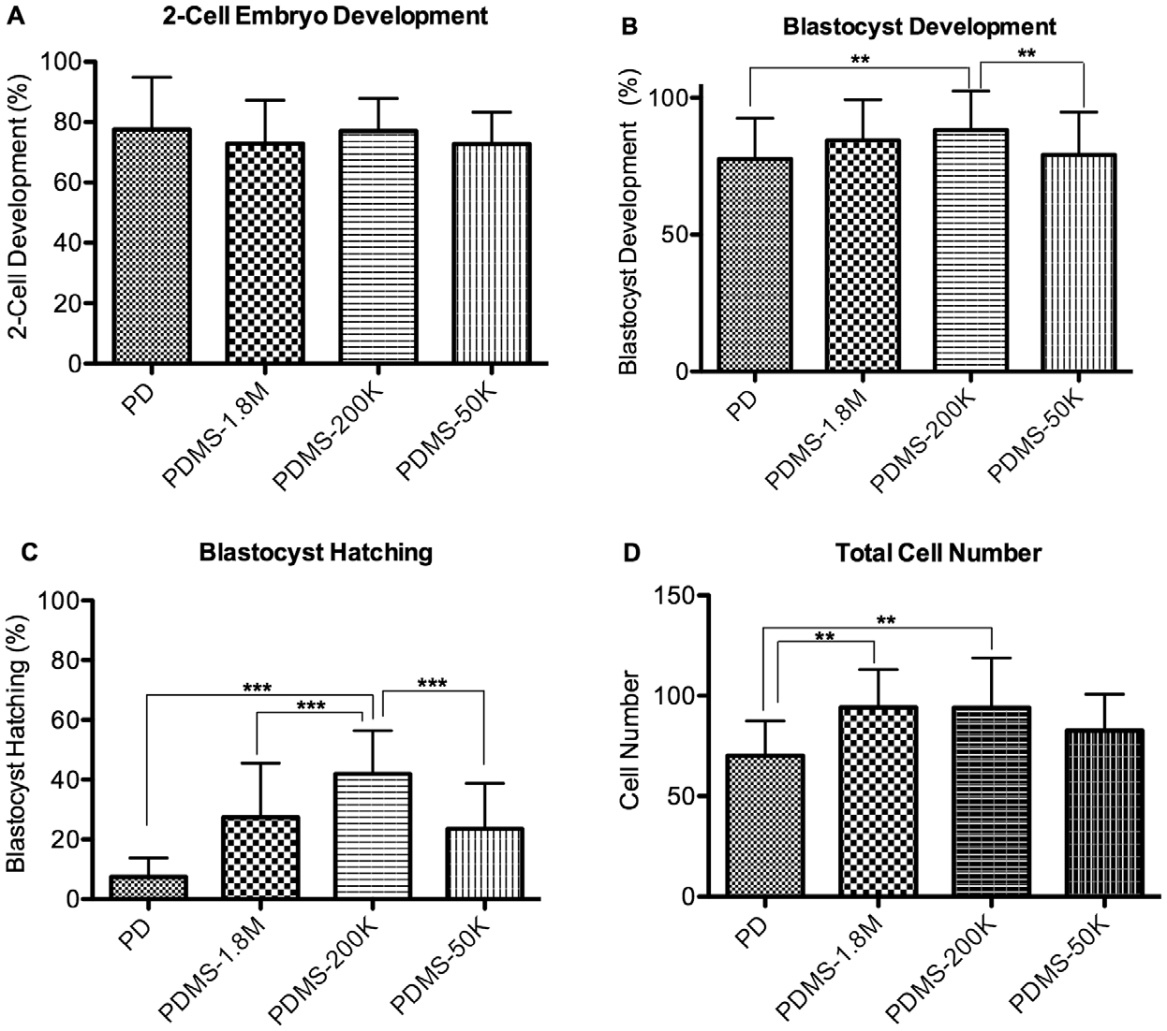
Effect of PDMS with varying stiffness or of polystyrene on zona intact embryos development. (**A**) Percent of zygotes progressing to the 2-cell stage (**B**) Percent of 2-cell embryos progressing to the blastocyst stage (**C**) Blastocyst hatching frequency. n = 14 replicates of ∼30 embryos each. (**D**) Total cell number. Error Bars: S.D. ** p<0.01; *** p<0.0001.

The PDMS stiffness did not affect the percentage of embryos developing to the 2-cell stage (PD 77±17%, PDMS-1.8 M 73±14%, PDMS-200K 77±11%, PDMS-50 K 73±11%); however more embryos (p<0.05) cultured on PDMS-200k developed to the blastocyst stage (88±14%) compared to embryos cultured on PD (78±15%), or PDMS-50K (73±15%) **(** **Figure 2A and B** **, Table S1)**. At E4.5 the blastocyst hatching frequency was significantly greater in the PDMS-200K group than in all of the other groups (PD 7.5±6.2%, PDMS-1.8 M 27±18%, PDMS-200 K 42±14%,PDMS-50 K 24±15%, p<0.05 **Figure 2C** **).**

To estimate whether increased blastocyst and hatching frequency was secondary to faster embryo development, we evaluated cell number. Total cell number in the blastocysts was significantly greater in the PDMS-200 K and PDMS-1.8 K embryos compared to the PD control, but cell number was no different compared to the very softest formulation of PDMS (PD 70±17, PDMS-200 K 94±24, PDMS-1.8 K 94±19, PDMS-50 K 83±18 p<0.01), implying more rapid development **(** **Figure 2D** **)**.

### Embryos Cultured on Soft (1 KPa) Collagen had an Increased 2 Cell, Blastocyst and Hatching Development and Increased TE Cell Number

**Figure 3:**
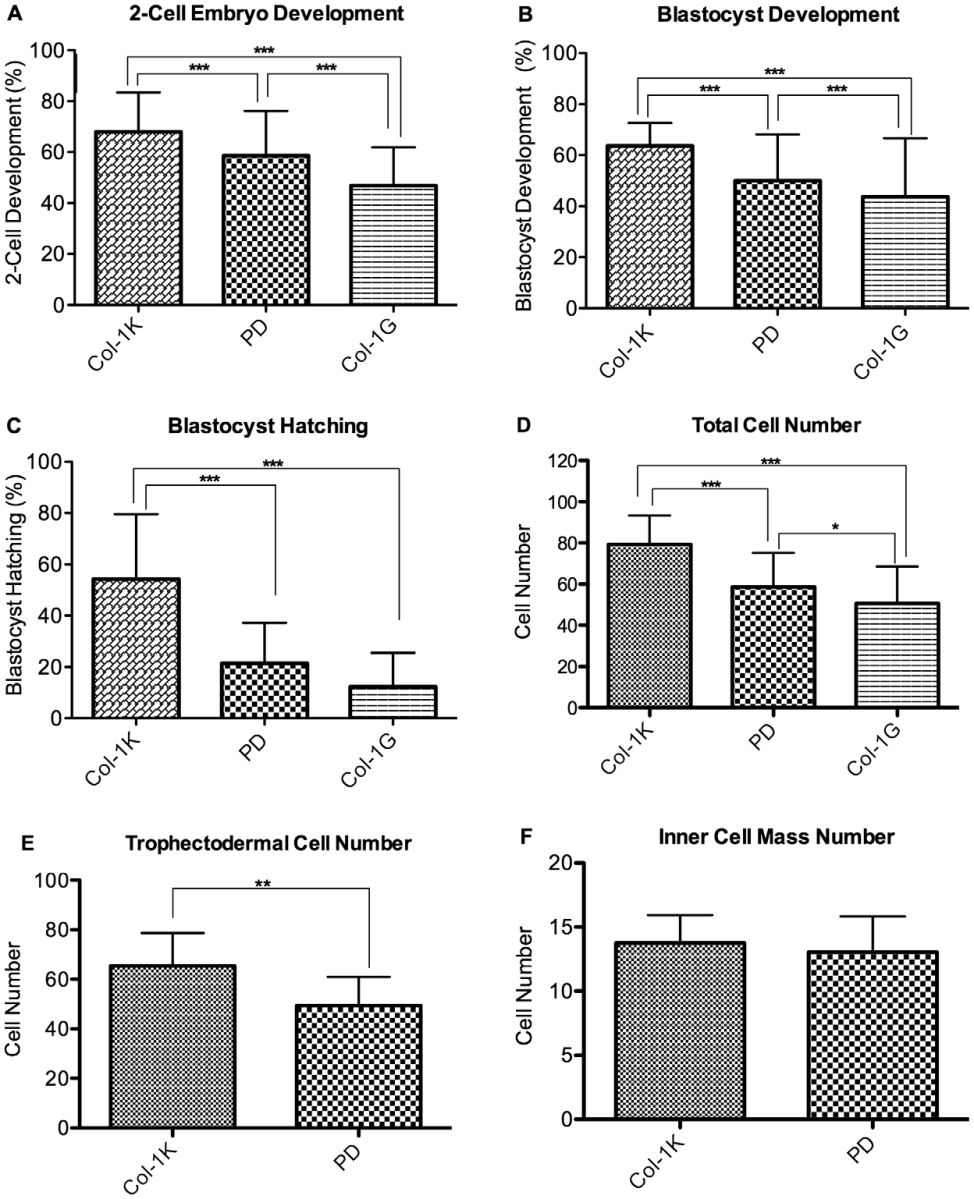
Effect of collagen or polystyrene on zona-intact embryo development. (**A**) Percent of zygotes progressing to the 2-cell stage (**B**) Percent of 2-cell embryos progressing to the blastocyst stage (**C**) Blastocyst hatching frequency. n = 14 replicates of ∼30 embryos in each replicate (**D**) Embryos cultured on collagen I have a greater number of total cells and (**E**) trophectodermal cells, but (**F**) equal number of inner-cell mass cells. n = 26 embryos. Error Bars: S.D. * p<0.05; ** p<0.01; *** p<0.0001.

As the softest formulation of PDMS yielded embryos with fewer cells and less frequent hatching, this appeared to be a suboptimal substrate. In the next series of experiments, zygotes were cultured on a soft collagen, which had a stiffness of 1 k Pa (Col-1k), similar to the one present in the fallopian tubes, or on two-dimensional collagen coated dishes, with a stiffness of 1 G Pa (Col-1G), which is similar to the stiffness of the PD. This last group was used to distinguish between the effects of substrate softness and a potential effect of collagen I itself. Importantly, the percentage of embryos developing to the 2-cell, blastocyst and hatching stage was significantly higher in embryos cultured on Col-1K, than on the stiffer substrates (PD or Col-1G, **Figure 3A, B, C** **, Table S1)**. In addition embryos cultured on Col-1G had statistically less frequent development to 2 cells and blastocysts compared to embryos cultured on PD.

We observed a significantly greater number of total cells in embryos cultured on Col-1k compared to the stiffer substrates (Col-1k (79±14), PD (59±17), Col-1G 50±18, p<.01, **Figure 3D**)**.** Blastocysts from the Col-1k and PD groups were analyzed further by differentially staining to distinguish inner cell mass (ICM) from the trophectodermal (TE) cells. Interestingly, the increased cell number in the Col-1 k group was due entirely to a greater number of TE (**Figure 3E**) as compared to controls (Col 1K, 65±13, PD 49±12) p<0.05). All of the blastocysts had a similar number of ICM cells (Col-1 K = 14±2 and PD = 13±3, ns **Figure 3F**).

### Embryos Cultured on Soft Collagen had Larger Placentae at E12.5

**Figure 4:**
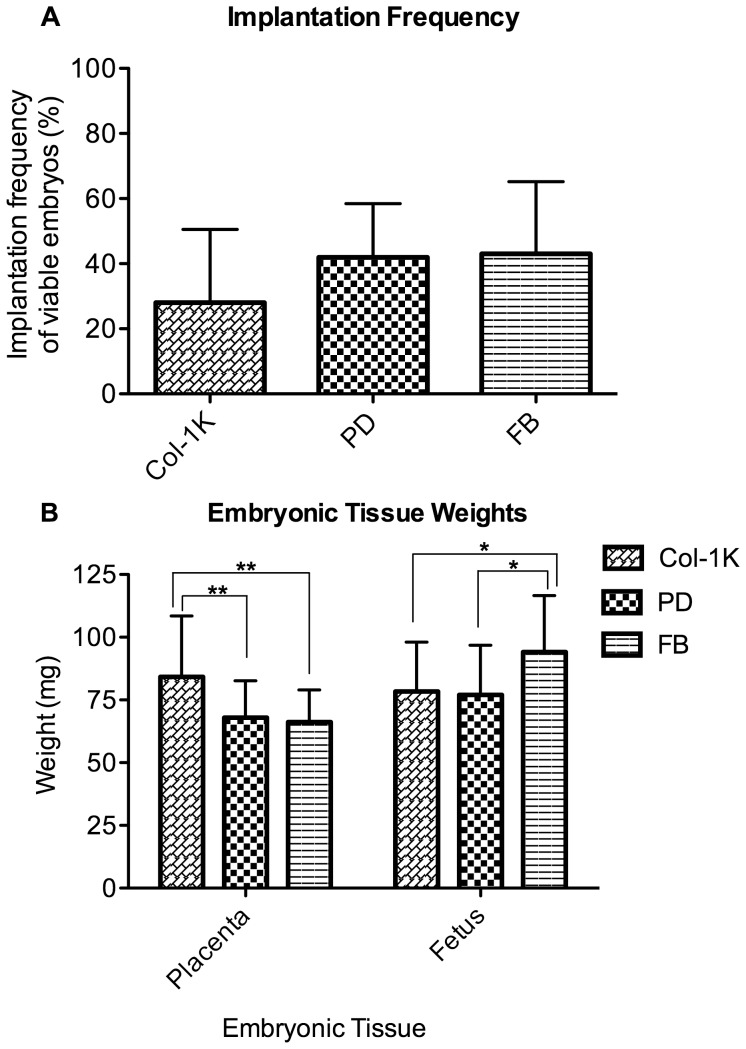
Effect of culture surface on implantation and fetal and placental wet weight. (**A**) Percent of live fetuses at E12.5 (# of viable fetuses/# embryos transferred). There was no significant effect of culture surface on implantation and survival to E12.5, (petri n = 9 litters, 72 embryos, collagen n = 9 litters 80 embryos, FB n = 9 litters, 68 embryos). (**B**) Placental weight. At E12.5, embryos that were cultured on collagen had significantly heavier placentas compared to both embryos cultured on polystyrene or *in vivo* transferred embryos (FB), Fetal weight was reduced in both cultured treatment groups. (PD n = 9 litters, 80 embryos, Col-1k n = 9 litters 80 embryos, FB n = 9 litters, 68 embryos) Error Bars: S.D. * p<0.05; ** p<0.01.

To test whether differences in embryo development translated into changes in implantation frequency and survival to E12.5, or to changes in fetal and placental weight, we transferred embryos cultured on Col-1k, and PD to recipient females. Additional control embryos were not cultured *in vitro*, but were merely flushed from the uterus of another mouse and transferred to a recipient (FB group). A total of 9 litters were generated and the results indicated that the frequency of implantation and survival of viable fetuses was not statistically different (28±22% Col-1k, 42±16% PD, and 43±22 FB ns, **Figure 4A**). Fetal weights were similar in both groups in which embryos were cultured, but these fetuses were significantly smaller than those in the FB group (Col-1k 78.4±19.7 mg, PD 77.1±19.7 mg, FB 94.1±22.5 mg, p<0.05, **Figure 4B**). In contrast, the weight of the placentas from embryos cultured on collagen I gels was significantly larger than those of either the PD or FB groups (Col-1k 84.2±14.7 mg, PD 67.9±24.2 mg, FB 66.2±12.9 mg, p<0.01, **Figure 4B**).

The presence of the GFP gene in embryos of one treatment group, allowed embryos from both culture groups to be transferred into the same recipient female. The GFP gene did not affect implantation/survival or placental or fetal weight.

### Expression of Mechano-responsive Genes was not Different after Culture on Col-1k

To test whether specific mechano-sensitive gene expression pathways were differentially activated in embryos cultured on Col-1k or PD, the expression of genes involved in response to mechanical cues (*ctgf* and *ankrd1*) [27] were examined. Trophectodermal differentiation markers (*cdx2, eomes*) [Bibr pone.0041717-Parks1] [28,29] and the proliferation-associated gene *cycd* were also measured.

Embryos cultured on Col-1k exhibited no significant differences in gene expression compared to those cultured on PD. However, compared to FB embryos, those cultured on Col-1k showed a significant 2-fold decrease in the expression of *cycd,* the proliferation marker (**Figure S2A**).

### Collagen Gel Supported Greater Development of Zona-free Embryos

As the effect of the substrate became more pronounced as the embryos approached hatching, we hypothesized that the zona pellucida (ZP) might buffer the embryo from the mechanical environment. To determine the forces that the embryo experienced as it touched the zona or the substrate, the weight of the embryo was measured. The embryo increased in radius as it developed, but the maximum velocity during free fall was relatively constant, indicating that the mass of the embryo had increased (**Table 1**).

**Table 1:** Distribution of forces on the zona pellucida, blastomeres or external environment.

Stage	Sed (s)	Free FallVelocity (mm/s)	Embryo weight(nN)	Radius (µm)^‡^	Zona stiffness (kPa)†	Indentation (nm)		
						3.7×10^9^ Pa (PD)	50×10^3 ^Pa (50∶1) PDMS	1×10^3^Pa (Col1k)
**Zygote (n = 13)**	12±1.5	0.90±0.10	0.88±.12	49±.37	22.3	21.47	27.46	792.01
**2 cell (n = 12)**	12±1.1	0.90±0.29	0.85±0.08	49±.64	13.8	28.92	34.03	776.15
**Morula (n = 8)**	11±1.6	0.96±0.1	0.87±0.11	47.5±2.1	1.88	112.27	115.07	821.70
**Blastocyst (n = 9)**	12±3.2	0.93±0.20	1.0±0.22	54.7±3.8	3.39	86.16	90.01	920.14

Individual blastomeres, especially those located most inferiorly, experience a significant force on the order of 1 nN. The extreme stiffness of the ZP relative to the weight of the embryo results only in minimal modification of the zona, when cultured in polystyrene. It will be the interface between the ZP and the external surface that will deform differently according to the characteristics of the external surface (F_Z-ex_). This is because only the softer of two materials will yield to pressure [45] (**Figure S3**). For example, the weight of the embryo on polystyrene will indent the ZP of 20–120 nm (0.1% to 0.7%) [Bibr pone.0041717-Murayama1] [34,46]. In contrast, when embryos are cultured on collagen gel, the collagen itself will yield (up to 1 µm) to the weight of the embryo and the ZP will not indent. Indentation is defined as the linear deformation experienced in the radial axis either by the embryo’s zona pellucida or by the substrate (collagen or PDMS). Sed: sedimentation time.

† *From* [*34*] *.*

The average mass was estimated to be on the order of 100 ng (zygote = 89.4±12.4 ng, 2-cell = 86.4±8.17 ng, morula = 88.47±11.0 ng, blastocyst = 115±9.87 ng), based on the Stokes’ law (**Table 1**)**.**

**Figure 5:**
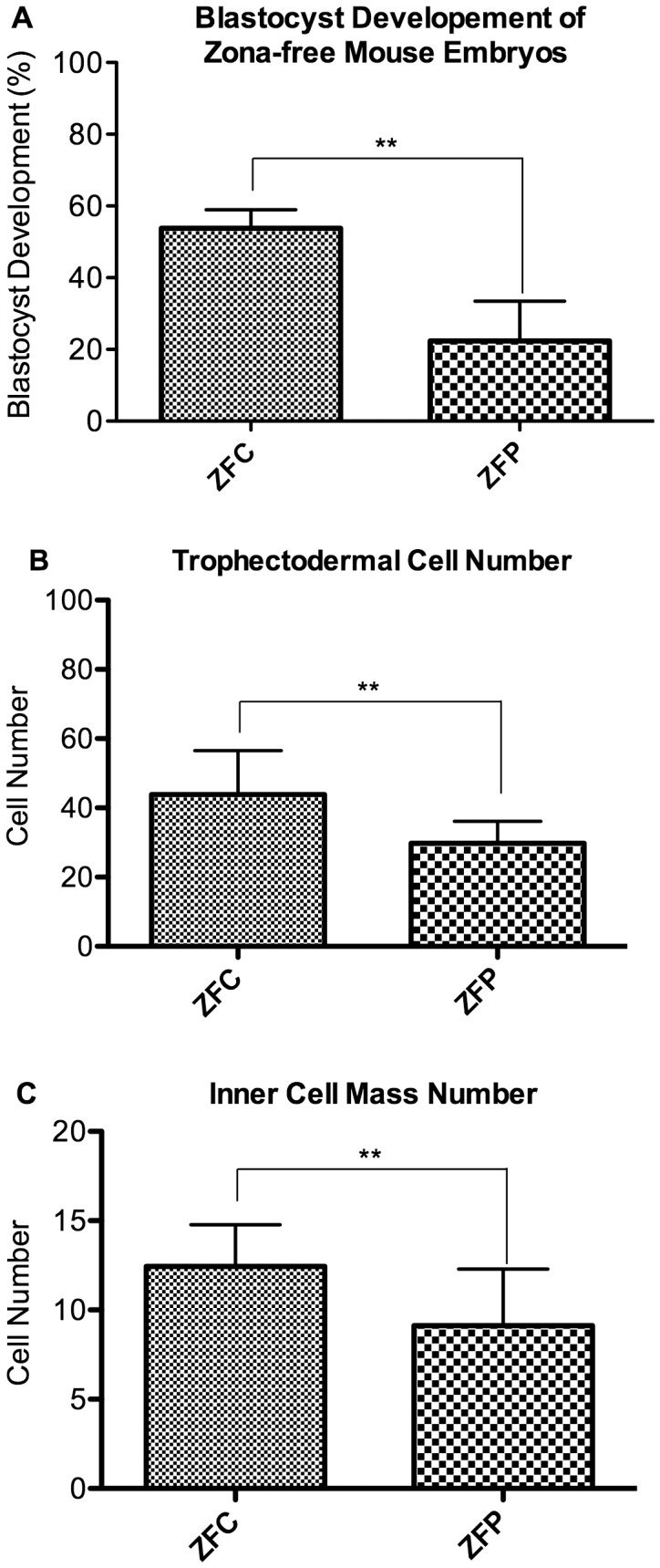
Effect of culture of zona-free embryos on collagen (ZFC) or polystyrene (ZFP). (**A**) Blastocyst development. Embryos cultured on softer surfaces developed into blastocysts more frequently than embryos cultured on polystyrene. (**B, C**) The number of TE (B) and ICM (C) cells of zona-free embryos. Zona-free embryos cultured on collagen I had a greater number of trophectodermal cells, but there was no significant difference in the number of cells in the inner cell mass. n = 15 embryos; Error Bars: S.D.** p<0.01.

To test how the substrate stiffness affected embryo development without the potential mechanical buffering effect of the ZP, the ZP were removed from 2-cell embryos (zona-free, ZF) and development was followed to the blastocyst stage. A greater percentage of ZF embryos cultured on collagen gel (ZFC) developed to the blastocyst stage than did ZF embryos cultured on plastic (ZFP) (ZFC 53±5%, ZFP 22±11%, **Figure 5A**). Moreover, ZFC blastocysts had a greater number of TE and ICM compared to ZFP embryos (TE: ZFC 44±13, ZFP 30±6, p<.01), **(** **Figure 5B** **)**; (ICM: ZFC = 12, ZFP = 9, p<.01) **(** **Figure 5C** **).** Zona-free blastocysts, regardless of culture surface, had significantly fewer TE and ICM and relative to their zona-intact counterparts (n = 65 p<.001). Zona free embryos cultured on collagen gel showed a significant, five-fold increase in *cdx2* (the TE marker) expression compared to zona free embryos cultured on PD (**Figure S2B**).

## Discussion

We conducted experiments to more closely replicate the mechanical environment that embryos encounter *in vivo* during their journey from the fallopian tube to the uterus. Our results indicate that embryos culture on a substrate with a stiffness approximating that of the fallopian tube and uterus is associated with more rapid preimplantation development and larger placentas at mid gestation. These findings have important implications and we believe that serious consideration should be given to developing technologies that allow culturing embryos in a physical environment that more closely mirrors the physiologic features of the genital epithelia.

First, we found that initial development to both 2-cell and blastocyst stage was dependent on the substrate stiffness, as the frequency of development from zygote to the 2-cell stage and from 2-cell to blastocyst were significantly different for embryos cultured on soft collagen gels (Col-1k) versus stiffer substrates (Col-1G or PD).

It is very likely that the zona pellucida functions as a shock absorber to dampen the effects of different mechanical environments when forces have profound effects in shaping future development [1] [Bibr pone.0041717-Wozniak1] [Bibr pone.0041717-Discher1] [4,12,30]. Therefore, the blastomeres in contact with the ZP will experience only the mechanical forces generated by the embryo weight (F_BZ_, **Figure S3**). Our assessment suggests that the embryo weight generates a force of approximately 1 nN, which is sufficient to affect developmental fate [12]. In fact, we calculated the wet weight of preimplantation embryos to be approximately 100 ng (**Table 1**), a value similar to the weight measured by others. Turner et al, in fact calculated that the dry weight of a blastocyst is 33 ng [31]. Considering that the embryo is composed for 80% water, these values are comparable.

Experiments using zona free embryos (**Figure 5**) confirm that the constant mechanical stress shielding provided by the zona is beneficial. In fact, zona free embryos cultured on polystyrene have a dramatically lower blastocyst frequency than that of zona-intact embryos (ZFP: 22% and PD = 78%, p<0.05). In addition, experiments on zona-free embryos indicate that the stiffness of the substrate has a greater effect; culture of zona free embryos on collagen improved not only the blastocyst development frequency (ZFC: 53%), but also the number of TE cells (ZFP: 30 cells and PD = 49 cells, p<0.05) and ICM cells (ZFP: 9 cells and PD = 12 cells, p<0.05) compared to zona-intact embryos. The beneficial effect of collagen served to blunt the negative effect of zona removal on the ICM (ZFC: n = 12 ICM cells). Culturing on soft collagen also increases the number of TE cells, but it does not completely restore TE cell number to that seen in control, zona-intact embryos (ZFC: n = 44; Col-1k: n = 65 TE cells).

A second finding was that softer environments appear more permissive for blastocyst hatching; furthermore, embryos cultured on softer environments had greater numbers of TE cell. These effects were consistent across different substrate types. On PDMS the effect was graded: culture on 200 KPa resulted in greater blastocyst development and hatching frequency than culture on 1.8 MPa or PD (10^9^Pa). It is unclear why culture on 50 KPa PDMS resulted in lower hatching frequency and lower TE cell number compared to 200 KPa. It is however known that there is often an optimal substrate stiffness that is unique for every material [Bibr pone.0041717-Discher1] [12,32].

It is unclear how the softness of the substrate affects hatching. An exhaustive explanation of the hatching process in different species is surprisingly missing [33]. Importantly, the ZP stiffness decreases by an order of magnitude from zygote to the morula and blastocyst stage, potentially facilitating the exit from the ZP [34]. The hatching process appears to be regulated by a variety of autocrine and paracrine molecules and by the presence of trophectodermal projections (TEPs). TEPs are cytoplasmic extensions of trophectodermal cells that protrude through the ZP into the extra-embryonic environment [Bibr pone.0041717-Gonzales1] [35,36]. During a 4-hour time window prior to hatching, TEPs reach a length of 17 microns and protrude and retract beyond the ZP [Bibr pone.0041717-Gonzales1] [35,36]. It is possible that the TEP detect physical and biochemical cues from the environment, specifically in this case its stiffness. The softer surface environment appears to be a pro-hatching signal.

While TEP could provide an explanation for the increase in hatching following culture in softer environment, it is unclear how to explain the increase in TE cell number noted in softer environment. Overall, it is believed that increases in TE and total cell number may give rise to better-quality in vitro-derived embryos: an Increase in TE cell number is observed following addition of folate, while culture in vitro or in stressful condition is associated with a decrease in TE cells [Bibr pone.0041717-Giritharan1] [8,37].

To further explore the mechanism of increase in TE cell number we tested a series of candidate genes. Surprisingly the gene expression data were not helpful, as expression of genes involved in trophectodermal differentiation (*cdx2, eomes*) [Bibr pone.0041717-Parks1] [28,29] or responsive to mechanical cues (*ctgf* and *ankrd1*) [27] was not different in zona intact embryo cultured in Col-1k or PD. Notwithstanding that embryos cultured in Col-1k have increased in TE cells number, these embryos had a paradoxical decreased expression of *cycd*, a marker of proliferation. It is unclear how to explain this last finding, although it is possible that measuring *cycd* level in isolated TE cells would provide different results.

Zona free embryos cultured on collagen have more TE cells and showed an increased expression of *cdx2.*

Overall the expression studies suggest that the selected genes are not reflective of the biochemical events that results from culturing cells on different substrate. Analysis of RhoA phosphorylation, a marker of activation of mechano-sensitive pathways, could provide an answer [38]. Unfortunately, the paucity of material does not permit embryonic testing.

Third, we found that *in vitro* embryos growing in softer (col-1K) and harder (PD) environments had equal frequencies of implantation and survival to E12.5 and equal fetal weights. However they had significantly larger placentas at E12.5 compared to embryos cultured on PD or FB controls. The lower fetal weight of *in vitro* embryos compared to *in vivo* embryos supports our prior data and confirms that *in vitro* generated embryo have a different in utero growth pattern compared to in vivo embryos [Bibr pone.0041717-DellePiane1] [9,39]. The larger placenta in the Col-1k is likely to be a result of the initial greater TE cell number. Overall the larger placentae following culture in soft environment might facilitate a compensatory growth in utero [39]. Indeed placental weight is positively correlated to bodyweight at term in a wide range of species and overall placental size directly affects the capacity for nutrient transfer via changes in the surface area for transport [40].

In this study we employed a newly devised method to apply protein based extracellular matrices, like collagen I, on hydrophobic material like polystyrene. This method included the fabrication of PDMS based wells that were activated via reactive ion etching and poly-d-lysine coating to form precisely sized 3D gels in wells for oil-immersed droplet culture. The method used here is highly modifiable to accommodate virtually any gel or well size.

One limitation of our study is that we tested few materials and stiffness in comparison with the myriad of those available. In addition, recapitulating the *in vivo* mechanical conditions goes beyond just modifying the surface on which the embryos rest. Ideally culture conditions should replicate all the in vivo mechanical conditions, including other externally applied forces, such as fluid shear, oviduct and uterine contractions, and electrostatic forces.

In summary, we found that embryos are mechano-sensitive and develop differently according to the specific stiffness of the environment. Our results suggest that culture on softer environments like collagen alters both pre-implantation and post-implantation development; current culture settings in an oil-immersed culture droplet on a polystyrene dish, are a far cry from the *in vivo* condition where the embryo is either suspended in a viscous tubal fluid or in contact with a uterine epithelium roughly six orders of magnitude softer than polystyrene. Future studies should include attempts to closely model the uterine environment, but should also better define the mechanical properties of the fallopian and uterine environment.

## Materials and Methods

### Animals

All experiments were approved by the Institutional Animal Care and Use Committee of the University of California, San Francisco and all animals were provided with nesting material and housed in cages maintained under a constant 12-h light/dark cycle at 21 to 23°C, with free access to standard chow (Pico-lab diet #5058∶23% protein; 22% fat and 55% carbohydrate) and tap water.

### Embryo Culture

Each experimental replicate consisted of 12 CF-1 female mice that were injected with 5 IU PMSG and 42–46 h later with 5 IU hCG. The females were then bred to 6 C57BL6/J males overnight. For experiments requiring embryo transfer, females were additionally mated to 6 C57BL6/J males homozygous for UBC-GFP (Jackson STOCK Tg(CAG-EGFP)B5Nagy/J): in this fashion control and experimental embryos could be transferred to the same foster mother. The following morning, zygotes were obtained from ampullae and cultured in potassium simplex optimization medium with amino acids (KSOM_AA_, MR-106-D, Millpore, Billerica, MA) on either polystyrene (PD group), collagen I with stiffness of 1 KPa (Col-1k or soft collagen) or collagen I with stiffness of 1 Gpa (Col-1G see below) to the blastocyst stage under 5% CO2 in humidified air at 37°C. The droplet to embryo ratio was held constant at roughly 1 embryo per 2 µl of KSOM_AA_.

Development to 2-cell, blastocyst, and hatching blastocyst was quantified by calculating the percentage of embryos that have proceeded to the corresponding developmental stage. In the case of hatching frequency, this is specifically defined as the percentage of embryos that are partially or completely hatched at 96 hours of *in vitro culture* or 108 hours after hCG administration.

### Removal of the Zona Pellucida

The zona-pellucida (ZP) was removed by immersing the embryos in acid Tyrode’s solution (Sigma), followed by immediate washing in KSOM [41]. Extra care was taken in minimizing the time and amount of manipulation of zona-free embryos.

### Embryo Transfer and Placental and Fetal Dissection

Recipient mice were generated by mating 6 weeks old CF1 females with CD1 vasectomized males; pseudo-pregnancy was determined by the discovery of a copulation plug the following morning (counted as embryonic day 0.5, or E0.5). Only late-cavitating blastocysts of similar morphology were selected for transfer. Ten to twelve embryos were transferred in each horn, on day 2.5 of pseudo pregnancy as previously described [9]. To control for maternal environment, embryos cultured on Col-1k or PD were transferred to the same recipient. In particular, for half of the experiments, GFP-expressing embryos were cultured on soft collagen and the wild-type embryos were cultured on PD. For the remaining half, the genotype of the embryos cultured on the two surfaces was reversed. Equal numbers of embryos from both surface types were transferred into the same recipient females. To control for the embryo transfer procedure and litter size, an additional control was added: blastocysts generated following natural mating were flushed out of the uterus and immediately transferred to recipient females (FB group) [9]. FB embryos were transferred to a distinct set of recipient mothers. Pregnant female mice at E12.5 were euthanized by CO_2_ inhalation and cervical dislocation. The fetuses were euthanized by decapitation. Dissection of the implantation sites was performed under an Olympus SZX9 dissecting microscope. The decidua was dissected away from the placenta. Immediately after dissection, each fetus and placenta was weighed individually. The number of GFP-expressing fetuses (and hence the number of embryos from a particular preimplantation condition) was determined by observing them with a Zeiss SteREO Discovery (Zeiss, Thornwood, NY, USA) fluorescence dissection microscope.

### Differential Staining of Trophectoderm and Inner Cell Mass

The number of trophectodermal (TE) and inner cell mass (ICM) cells from expanded blastocysts were determined by a dual nuclear staining method as previously described [8]. Briefly, the blastocysts were exposed to 1% Triton ×100 (Sigma-Aldrich CO., St. Louis, MO, USA) in minimum essential medium (MEM; Invitrogen Corporation, Grand Island, NY, USA) for 3–5 seconds, washed 3–5 times in MEM+polyvinylpyrrolidone (PVP; Sigma-Aldrich CO.) and incubated in MEM+PVP containing 100 µg/ml propidium iodide (Sigma-Aldrich CO.) for 30 seconds. The embryos were then washed 3–5 times in MEM+PVP and fixed overnight in 100% ethanol containing 25 µg/ml of bizbenzamide (Sigma-Aldrich CO.). The embryos were mounted on a clean glass slide using glycerol and kept in a dark chamber until observed under fluorescence light using a Leica microscope (Model DMRB, Leica Microsystems AG, Wetzlar, Germany).

The embryos were observed under fluorescence microscope and the numbers of ICM (blue) and TB (red) cells were counted at 200 X magnification. The staining procedure was repeated three times per treatment group with different sets of blastocysts (n≥30).

### Assessment of the Forces Experienced by the Embryo

We developed a simple method of identifying the weight of the embryo at various stages. Briefly, we estimated the weight of an embryo by measuring the velocity of the falling embryo in a column of liquid of height 1.1 cm, using Stokes’ law (F_d_ = 6πµRV_s_, where R is the radius, μ is the dynamic viscosity of water at 37**°**C (Pa*s), and V_s_ is sedimentation velocity of the embryo) for drag force and approximating the embryo to a sphere with low Reynolds number [42]. The drag force is equal to the weight of the embryo at terminal free fall velocity. Free fall velocity can be calculated by measuring the time required for an embryo to fall the entire height of the column. The time is calculated from the time the embryo is released on the top of the column of fluid to the time it reaches the bottom of the dish; the time of arrival is visualized by focusing on the bottom of dish at high magnification (40X). The radius of embryos at different stages of development was measured using an inverted microscope.

Based on these calculations, we found that the mass of a mouse preimplantation embryo is on the order of approximately 100 ng. Masses were translated into force (Newton), according to Newton’s second law (F = ma; N = kg×m/s^2^) 100 ng ∼ 1 nN (1 g = 9.8 mN).

Mechanical properties of collagen gels, PDMS, and polystyrene were estimated from published values [Bibr pone.0041717-LopezGarcia1] [Bibr pone.0041717-Brown1] [26,43,44].

### Collagen Gel Preparation

Here, we invented a methodology by which we were able to fabricate 6 mm diameter 3D Collagen I gels (BD Biosciences, Bedford, MA, USA), which could be used for oil immersed droplet culture. Essentially, PDMS was employed to fabricate wells with a depth of 1 mm and diameter of 6mm to support the droplet and gel structure. In order to ensure the collagen gel adhered to the PDMS, only the bottom surface of the well was exposed to reactive-ion etching at 200 W for 30 s (Plasmaline, PlasmaTek, Carson City, NV). This treatment rendered the PDMS surface highly ionized and hydrophilic. A mask made of a second layer of PDMS with 6 mm holes was necessary to shield the area bordering the wells in order to allow embryo droplet formation. This second layer of PDMS was then placed on top of a sheet that was similarly exposed to etching and bonded. This effectively produced wells of 6 mm thickness bordered by PDMS that was protected from etching **(Figure S1)**. To promote the longevity of the hydrophilic surface we applied a coating of Poly-D-Lysine. 1 mg/ml (Sigma-Aldrich CO.) before subsequent application of 25 ul of collagen I gel. The gel formed is then highly resilient and was cleaned 3 times with KSOM.

Since a collagen gel can potentially provide both mechanical and biochemical cues we compared the culture effects of the soft collagen gel to a commercially available collagen coated poly-styrene dish (Hard Collagen, BioCoat™ BD Biosciences, Bedford, MA, USA Cat# 345456). The hard collagen environment represents a 2-dimensional thin layer, and should more closely approximate the mechanical properties of the petri dish, but recapitulate the biological aspects of the gel collagen environment.

### PDMS Gel Preparation

PDMS gel was formed by mixing a 10∶1 w:w ratio of the silicone elastomer base with the crosslinking agent Sylgard 184 (Dow Corning, Midland MI), as described [43]. Mixtures of 10∶1, 25∶1 and 50∶1 elastomer base: curing agent were thoroughly mixed and directly coated onto petri dishes. The PDMS coated petri dishes were first degassed for 20 minutes and then transferred to an oven at 60°C to cure overnight. Embryos were cultured on PDMS surfaces with 3 stiffnesses: 1.8 MPa (PDMS-1.8 M), 200 KPa (PDMS-200K), 50 KPa (PDMS-50K) as well as on the control petri dish (PD, 1 GPa).

### Real Time PCR

Blastocysts developed *in vivo* and *in vitro* (KSOM/AA) were collected as described above. At least 5 blastocysts were collected from a single experiment and constitute a single replicate. Total RNA was isolated with RNeasy Protect Mini Kit and then treated with RNase-free DNase according to the manufacturer’s instructions (Qiagen, Valencia, CA, USA). Concentration and purity of total RNA was assessed by electrophoresis and by using Nanodrop (Thermo Scientific, Wilmington, DE, USA). Reverse transcription (RT) was carried out using a commercially available first strand cDNA synthesis kit (Bio-Rad Laboratories, Hercules, CA, USA). The RT reactions were then performed according to the kit manufacturer’s protocol and using 0.25 embryo equivalent. Quantifying the nucleic acid present in a sample that contained no reverse transcriptase in the RT step served as a control for the presence of genomic DNA contamination in the sample.

Real-time quantitative PCR was performed using SYBR green PCR supermix (Bio-Rad Laboratories). Primer sequences for the genes analyzed are the following, from *Ctgf* Forward: CCCCCGCCAACCGCAAGAT Reverse: CACCGACCCACCGAAGACACAGG, *Cdx2* Forward: GCGGCTGGAGCTGGAGAAGGAGTT Reverse: CGGCGGCTGTGGAGGCTGTTGT, *Ankrd1* Forward: GCTGCGCTGGAGAACAAACTG Reverse: AGCCTCCATTAACTTCTCCACGAT, *Cycd1* Forward: GCAGCCCCAACAACTTCCTCTCCT Reverse: CCGGGCTGGCTCCTTCCTCTTT, *Eomes* Forward: GCGGCCGGGTCTTGTGGAGGATTG Reverse: TGTAGTGGGCGGTGGGGTTGAGTC All samples were normalized to levels of Histone H2A Forward: ACATGGCGGCGGTGCTGGAGTA Reverse: CGGGATGATGCGCGTCTTCTTGTT. Duplicates were used for each real-time PCR reaction while a no-template control was made in all runs using water in place of cDNA. For each group, real-time PCR was repeated twice while real-time PCR data was analyzed within the log linear phase of the amplification curve obtained for each probe/primer using the comparative threshold cycle method (Bio-Rad Laboratories). Thermal cycle conditions used as follows: 40 cycles of 94°C for15 sec, 55°C for 30 sec, and 72°C for 30 sec using cDNA (0.25 embryo equivalent) in a final reaction volume of 25 µl.

### Statistics

All statistics were performed using SAS (version 9.2, 64-bit). For dichotomous variables comparisons such as whether an embryo progressed to the 2-cell, blastocyst, hatching blastocyst stage or whether an embryo survived to E12.5 or not, logistic regression was used. For analysis of cell number, gene expression, and placental and fetal weight, ANOVA was used. Pairwise comparisons were performed using Bonferroni adjustment. P-values less than 0.05 were considered significant. Results are expressed as mean ±SD.

## Supporting Information

Figure S1 **Method used to create wells for culturing embryos on collagen.** PDMS (10∶1) was first layered onto a polystyrene dish to create a uniform, flat gel. After the first layer was cured, a second layer of PDMS, also pre-cured and pre-cut with holes, was laid on top of it. The two layers were then permanently bonded by exposing them into a reactive ion etcher. To promote the longevity of the hydrophilic surface we applied a coating of Poly-D-Lysine (0.1 mg/ml) before subsequent application of 25 ul of collagen I (3 mg/ml) gel. See material and methods for additional details.
(TIF)

Figure S2 **Gene expression in** ***in vivo*** **embryos or embryos cultured on collagen or polystyrene. A.** Gene expression changes of selected genes assessed by real time RT PCR: *cycd* (proliferation), *cdx2, Eomes* (trophectoderm lineage commitment*)* and *ankrd1, ctgf* (mechanotransduction). To facilitate comparisons expression in the FB was normalized to 1. Embryos cultured on collagen I have significantly decreased expression of *cycd* compared to FBs (p = .03), implying decreased mitoses. All other comparisons of gene expression were not significantly different. **B.** Zona-free embryos cultured on collagen gels exhibited a five-fold increase in expression of *cdx2* relative to zona free embryos cultured on standard polystyrene petri dishes. n = 3 replicates of 5 embryos. Error Bars: S.D.* p<0.05
(TIFF)

Figure S3 **Distribution of forces in the preimplantation embryo. (A)** F_Z-EX_ and F_B-Z_ represent net forces on the zona and on the blastomeres. F_Z-EX_ is defined as the net force operating on the ZP because of the extra-embryonic environment; F_B-Z_ indicates the net force acting on the blastomeres and caused by the ZP. While both F_B-Z_ and F_Z-EX_ are dependent upon the embryo stage and size, the manner in which they are distributed can be drastically different according to the stiffness characteristics of the environment. (**B)** The ZP is flattened because of the contact with the polystyrene. (**C)** The collagen is compressed by the weight of the embryo, while the ZP shows no changes in thickness. In both conditions the blastomeres will experience the same total force F_B-Z_.
(TIF)

Table S1 **Effect of Culture Environment on Preimplantation Embryo Development.** Tabulated distribution of embryo development after *in vitro culture* for 96 hours in substrates of different stiffness.
(DOCX)
