# Supplementary figures and images for: Effect of Substrate Stiffness on Early Mouse Embryo Development
Source: PLoS One. 2012 Jul 31;7(7):e41717. doi: 10.1371/journal.pone.0041717 (PMC3409240; doi:10.1371/journal.pone.0041717)

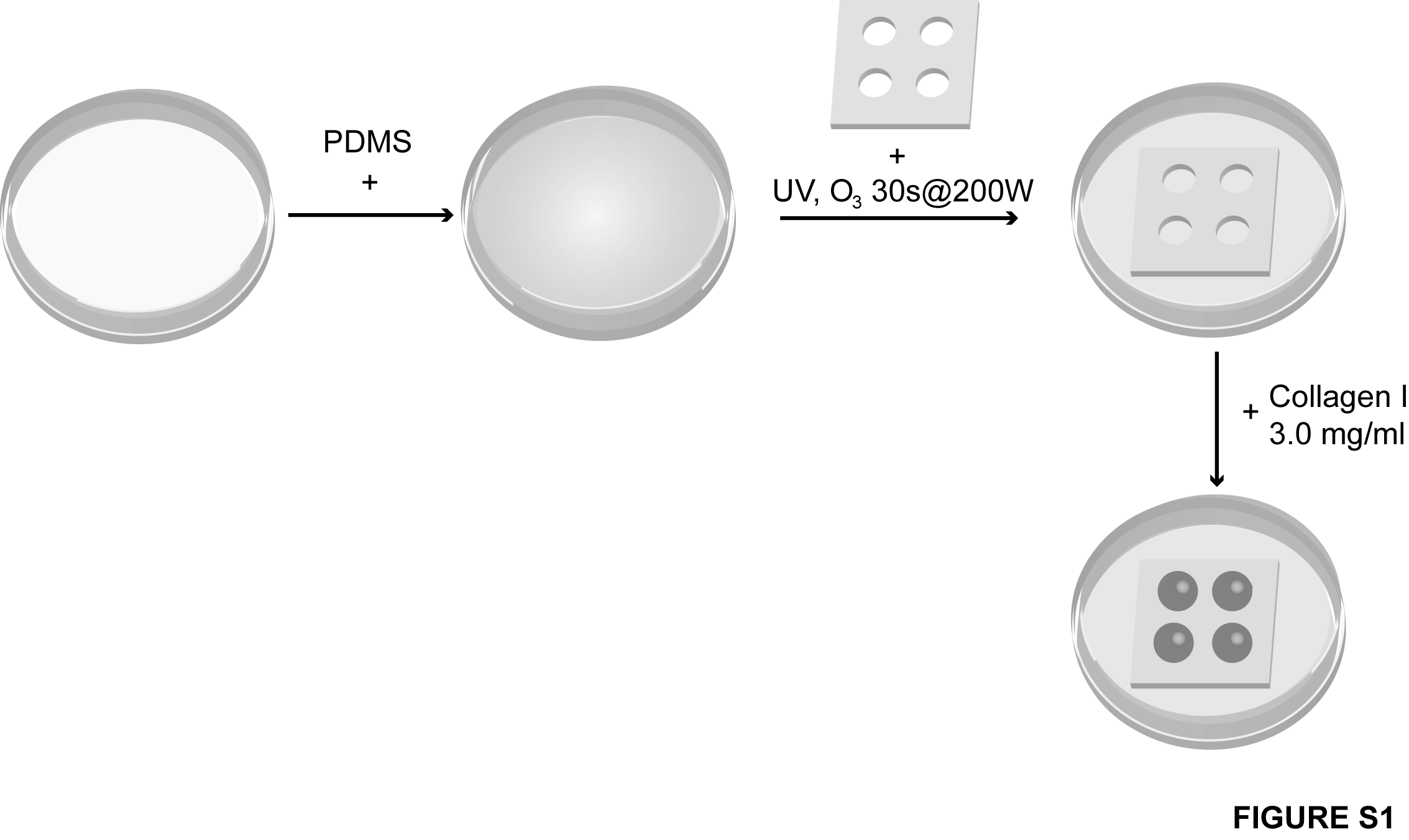

Supplement: Figure S1 — Method used to create wells for culturing embryos on collagen. PDMS (10∶1) was first layered onto a polystyrene dish to create a uniform, flat gel. After the first layer was cured, a second layer of PDMS, also pre-cured and pre-cut with holes, was laid on top of it. The two layers were then permanently bonded by exposing them into a reactive ion etcher. To promote the longevity of the hydrophilic surface we applied a coating of Poly-D-Lysine (0.1 mg/ml) before subsequent application of 25 ul of collagen I (3 mg/ml) gel. See material and methods for additional details. (TIF) [file pone.0041717.s001.tif]

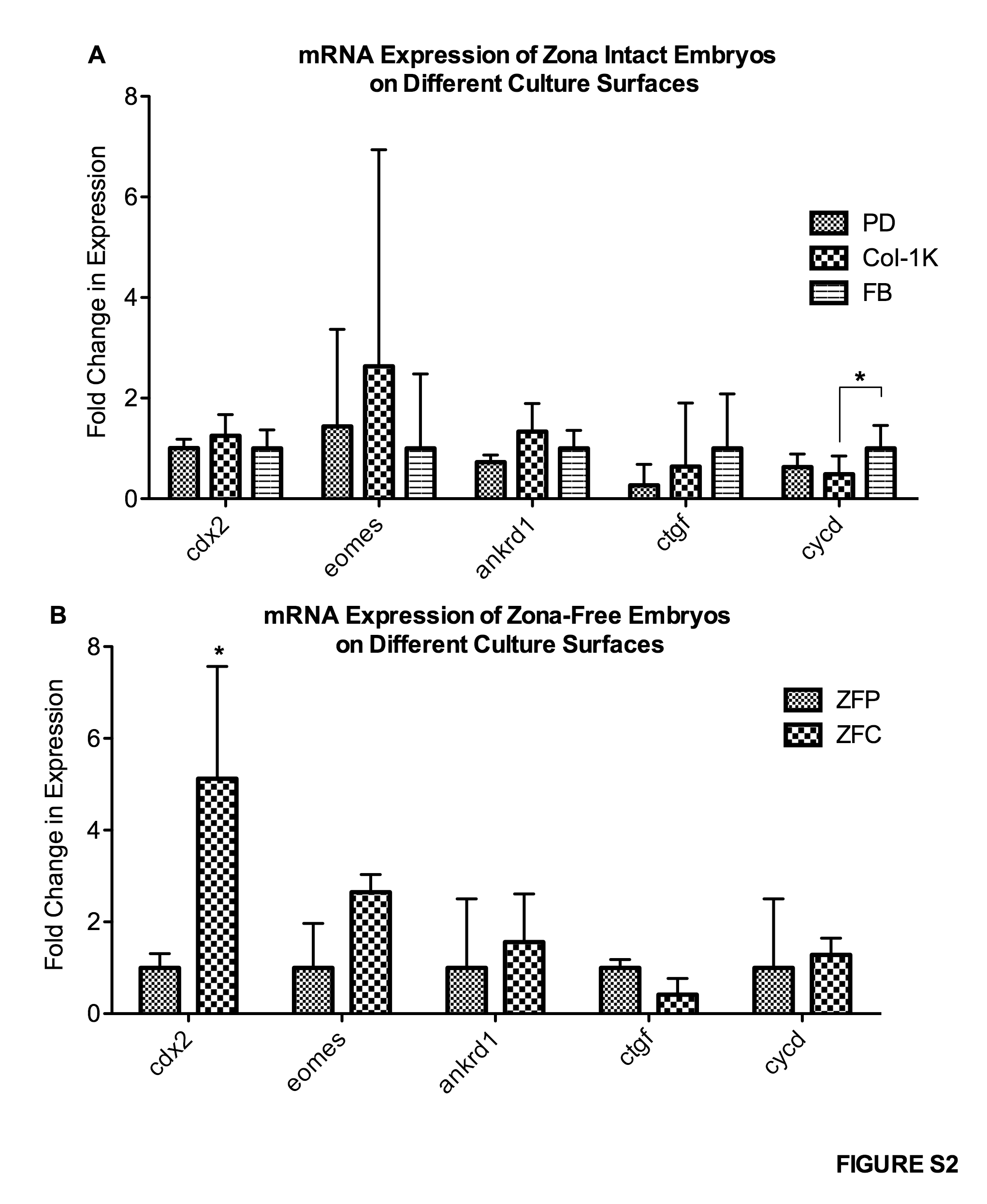

Supplement: Figure S2 — Gene expression in in vivo embryos or embryos cultured on collagen or polystyrene. A. Gene expression changes of selected genes assessed by real time RT PCR: cycd (proliferation), cdx2, Eomes (trophectoderm lineage commitment) and ankrd1, ctgf (mechanotransduction). To facilitate comparisons expression in the FB was normalized to 1. Embryos cultured on collagen I have significantly decreased expression of cycd compared to FBs (p = .03), implying decreased mitoses. All other comparisons of gene expression were not significantly different. B. Zona-free embryos cultured on collagen gels exhibited a five-fold increase in expression of cdx2 relative to zona free embryos cultured on standard polystyrene petri dishes. n = 3 replicates of 5 embryos. Error Bars: S.D.* p<0.05 (TIFF) [file pone.0041717.s002.tiff]

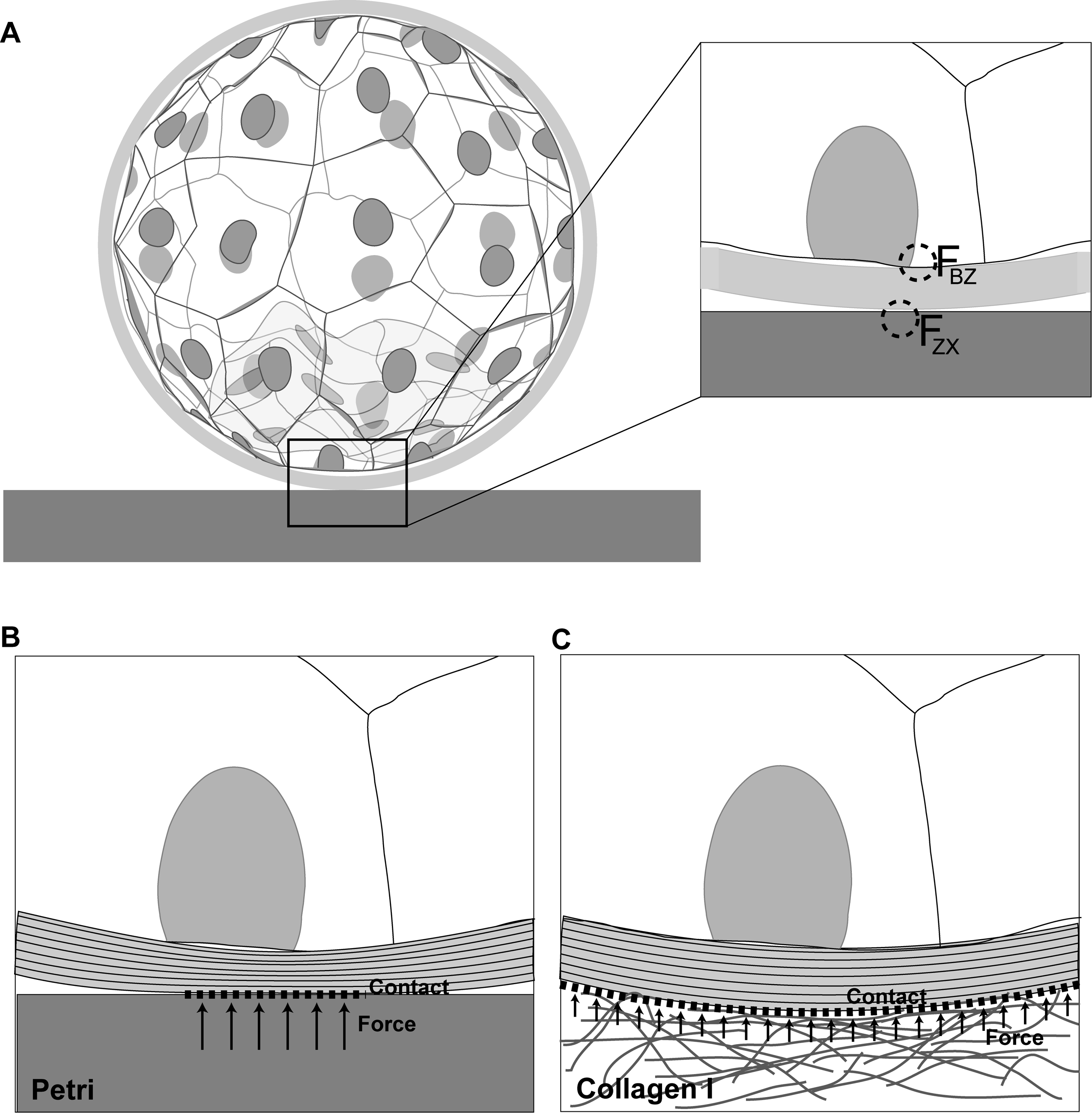

Supplement: Figure S3 — Distribution of forces in the preimplantation embryo. (A) FZ-EX and FB-Z represent net forces on the zona and on the blastomeres. FZ-EX is defined as the net force operating on the ZP because of the extra-embryonic environment; FB-Z indicates the net force acting on the blastomeres and caused by the ZP. While both FB-Z and FZ-EX are dependent upon the embryo stage and size, the manner in which they are distributed can be drastically different according to the stiffness characteristics of the environment. (B) The ZP is flattened because of the contact with the polystyrene. (C) The collagen is compressed by the weight of the embryo, while the ZP shows no changes in thickness. In both conditions the blastomeres will experience the same total force FB-Z. (TIF) [file pone.0041717.s003.tif]
