# Supplementary material for: Effect of Substrate Stiffness on Early Mouse Embryo Development
Source: PLoS One. 2012 Jul 31;7(7):e41717. doi: 10.1371/journal.pone.0041717 (PMC3409240; doi:10.1371/journal.pone.0041717)
Supplement: Table S1 — Effect of Culture Environment on Preimplantation Embryo Development. Tabulated distribution of embryo development after in vitro culture for 96 hours in substrates of different stiffness. (DOCX) [file pone.0041717.s004.docx]

| Culture Surface | Number of Zygotes | Number of 2-cell embryos  (24 hours) | Number of Non-blastocysts  (96 hours) | Number of Blastocysts  (96 hours) | Number of Hatching Blastocysts  (96 hours) | Zygote to 2-cell  (%)  (mean±SD) | 2-cell to Blastocyst  (%)  (mean±SD) | Blastocyst to Hatching Blastocyst (%)  (mean±SD) |
| --- | --- | --- | --- | --- | --- | --- | --- | --- |
| PD | 266 | 189 | 29 | 112 | 11 | 77±17 | 78±15 | 7.5±6.2 |
| PDMS-1.8M | 273 | 191 | 18 | 114 | 25 | 73±14 | 84±15 | 27±18 |
| PDMS-200K | 278 | 207 | 22 | 109 | 55 | 77±11 | 88±14^*^ | 42±14^*^ |
| PDMS-50K | 253 | 144 | 47 | 104 | 18 | 73±11 | 73±15 | 24±15 |

* Values with different superscripts in each column differ significantly (P<.01).

§ *(CF-1xB6D2)*

| Culture Surface | Number of Zygotes | Number of 2-cell embryos  (24 hours) | Number of Non-blastocysts  (96 hours) | Number of Blastocysts  (96 hours) | Number of Hatching Blastocysts  (96 hours) | Zygote to 2-cell  (%)  (mean±SD) | 2-cell to Blastocyst  (%)  (mean±SD) | | Blastocyst to Hatching Blastocyst (%)  (mean±SD) |
| --- | --- | --- | --- | --- | --- | --- | --- | --- | --- |
| PD | 850 | 541 | 226 | 269 | 62 | 59±18 | 50±18 | 21±16 | |
| Col-1K | 696 | 452 | 187 | 303 | 163 | 68±15^*^ | 64±9.1^*^ | 54±25^*^ | |
| Col-1G | 578 | 280 | 192 | 131 | 43 | 47±15^†^ | 44±23^†^ | 12±13^†^ | |

*,† Values with different superscripts in each column differ significantly (P<.01).

§ (*CF-1xC57BL6/J)*

| Treatment Group | Number of 2-cell embryos  (24 hours) | Number of Non-blastocysts  (96 hours) | Number of Blastocysts  (96 hours) | 2-cell to Blastocyst  (%)  (mean±SD) |
| --- | --- | --- | --- | --- |
| ZFP | 135 | 105 | 30 | 22±11 |
| ZFC | 129 | 59 | 70 | 53±5.0^*^ |

* Values with different superscripts in each column differ significantly (P<.01).

§ (*CF-1xC57BL6/J)*
